# Multi-Modal Integration of EEG-fNIRS for Brain-Computer Interfaces – Current Limitations and Future Directions

**DOI:** 10.3389/fnhum.2017.00503

**Published:** 2017-10-18

**Authors:** Sangtae Ahn, Sung C. Jun

**Affiliations:** ^1^School of Electronic and Electrical Engineering, Kyungpook National University, Daegu, South Korea; ^2^School of Electronics Engineering, Kyungpook National University, Daegu, South Korea; ^3^School of Electrical Engineering and Computer Science, Gwangju Institute of Science and Technology, Gwangju, South Korea

**Keywords:** multi-modal integration, electroencephalography (EEG), functional near-infrared spectroscopy (fNIRS), brain-computer interface (BCI)

## Abstract

Multi-modal integration, which combines multiple neurophysiological signals, is gaining more attention for its potential to supplement single modality’s drawbacks and yield reliable results by extracting complementary features. In particular, integration of electroencephalography (EEG) and functional near-infrared spectroscopy (fNIRS) is cost-effective and portable, and therefore is a fascinating approach to brain-computer interface (BCI). However, outcomes from the integration of these two modalities have yielded only modest improvement in BCI performance because of the lack of approaches to integrate the two different features. In addition, mismatch of recording locations may hinder further improvement. In this literature review, we surveyed studies of the integration of EEG/fNIRS in BCI thoroughly and discussed its current limitations. We also suggested future directions for efficient and successful multi-modal integration of EEG/fNIRS in BCI systems.

## Introduction

Electroencephalography (EEG) can record electrical changes induced by extra- and intra-cellular electric currents associated with neuronal activity passively using scalp electrodes. EEG is one of the most common techniques used to investigate the brain’s underlying mechanisms and is used widely in a variety of neuroscience fields. To extract features from EEG data, temporal and spectral analysis are used after elaborate pre-processing steps, such as removing artifacts from eye-blinking or muscle movement. Event-related potentials, according to average in response to time-locked repetitive sensory stimuli and rhythmic neural oscillations caused by interactions between neurons, are conventional temporal and spectral features, respectively. One of the advantages of EEG is its higher temporal resolution, but it has a lower spatial resolution and lower signal-to-noise ratio attributable to the inherent low conductivity of the skull than does functional magnetic resonance imaging (fMRI), which relies on the fact that cerebral blood flow and neural activations are associated closely.

Measuring cerebral blood flow can give us significant information necessary to investigate brain dynamics as well as electrical activity. Similarly, functional near-infrared spectroscopy (fNIRS) is a portable technique suitable for cost-effective measurement of cerebral blood flow in the brain (Villringer et al., 1993). fNIRS is based on in the amount of measure photons using light in the near-infrared range (700–900 nm). Quantification of chromophore concentration and its relative changes between two different frequencies of infrared are fundamental processes that can be explained by the modified Beer-Lambert’s law (Delpy et al., 1988). Because oxygenated (HbO) and deoxygenated hemoglobin (HbR) have characteristic optical properties in the visible and near-infrared light range, the changes in concentration of these molecules are the main features of fNIRS. Although this technique has attracted attention because of its ability to measure hemodynamic responses, similar to fMRI, the problems of low spatial- and depth-resolution still remain.

Overall, integration of EEG and fNIRS can provide us with two different sources of information about the brain, electrical activities and hemodynamic responses; this integration has the advantages of non-invasiveness, portability, and cost-effectiveness, among others. For these reasons, one of the primary targets for their integration is brain-computer interface (BCI). BCI using EEG has been used widely since Vidal first introduced a direct BCI (Vidal, 1973). Intendix, Corp.^1^ commercialized the first visual EEG-BCI system with high accuracy and reliability. Recently, fNIRS-BCI has emerged as a new potential approach (Naseer and Hong, 2013, 2015). fNIRS can measure HbO and HbR in the superficial layers of the human cortex, and may be less susceptible to electrical noise and movement artifacts. Thus, multi-modal integration of EEG and fNIRS has received attention as a new BCI paradigm.

However, improvement in BCI performance using multi-modal integration is still in its infancy. The modest improvement may be caused by the lack of computational approaches to combine the two different features. One obstacle in the development of novel computational approaches to combine two different features is the mismatch in their temporal resolution and inherent delays in hemodynamic responses, which can disrupt the simultaneous integration of features. In addition, a mismatch in recording locations between EEG and fNIRS attributable to technical problems may hinder improvement in BCI performance and interpretation of neurophysiological findings from the two different locations. In this literature review, we reviewed reports of multi-modal integration of EEG-fNIRS in BCI (summarized in **Table 1**), and discuss its current limitations. Further, we suggest future directions for successful multi-modal integration of EEG-fNIRS in BCI.

**Table 1:** Summarized findings in multi-modal integration of EEG-fNIRS (EEG, electroencephalography; fNIRS, functional near-infrared spectroscopy; HbO/HbR, concentration changes of oxygenated/deoxygenated hemoglobin; ERD, event-related desynchronization; SSVEP, steady-state visual evoked potential).

Reference	Regions of recording	Task	Feature	Major findings
Fazli et al., 2012	Frontal, sensorimotor, and parietal	Motor execution and imagery	EEG: band power; fNIRS: HbO and HbR	Classification accuracies in motor execution and imagery for 14 healthy subjects improved significantly using simultaneous EEG and fNIRS compared to signal modality.
Kaiser et al., 2014	Sensorimotor	Motor imagery	EEG: band-power; fNIRS: HbO and HbR	EEG-based feedback training increased HbO in fNIRS and a stronger ERD in the beta band were achieved in low BCI performers (<70%).
Khan et al., 2014	EEG: sensorimotor; fNIRS: prefrontal	Mental arithmetic and motor imagery	EEG: peak amplitudes; fNIRS: HbO and HbR	Mental arithmetic and hand tapping were decoded from fNIRS and EEG signals, respectively. High classification accuracies (>80%) were obtained in four tasks.
Tomita et al., 2014	Occipital	Visual attention to flickering visual stimuli	EEG: SSVEP; fNIRS: HbO and HbR	fNIRS signal in the occipital region was used as a brain switch to activate the SSVEP BCI. Improvement in SSVEP classification and a reduction of error rates for 13 subjects were achieved.
Morioka et al., 2014	EEG: whole scalp; fNIRS: parietal and occipital	Spatial attention	EEG: alpha and beta spectral power; fNIRS: HbO	EEG-fNIRS decoder using cortical current estimation yielded performance that was significantly better than with decoding methods based on EEG sensor signals alone.
Putze et al., 2014	EEG: whole scalp; fNIRS: temporal and occipital	Visual and auditory perception	EEG: event-related potential and power spectral density; fNIRS: HbO and HbR	Subject-dependent approach achieved a high classification accuracy (>90%) in discriminating between visual and auditory perception and an idle state.
Yin et al., 2015	Sensorimotor	Motor imagery	EEG: time-frequency-phase feature; fNIRS: HbO and HbR	Simultaneous EEG-fNIRS features for decoding motor imagery of both force and speed of hand clenching achieved improved classification accuracy compared to signal modality.
Koo et al., 2015	Sensorimotor	Motor imagery	EEG: alpha-band power; fNIRS: HbO	A new system to block leaking light from fNIRS was developed. An online self-paced motor imagery was performed using EEG-fNIRS and fNIRS signals were used as a brain switch. The system has a true positive rate of 88%, a false positive rate of 7% with an average response time of 10.36 s
Buccino et al., 2016	Sensorimotor	Motor execution	EEG: band-power; fNIRS: HbO and HbR	Newly developed slope indicators, which are used to detect immediate changes, decreased the delays of peak classification accuracy up to 2 s in fNIRS.
Ahn et al., 2016	EEG: whole scalp; fNIRS: prefrontal	Simulated driving	EEG: alpha/beta power fNIRS: HbO	A new feature combination method was proposed based on normalization of each feature. EEG-fNIRS feature combination distinguished clearly between well-rested and sleep-deprived conditions.
Nguyen et al., 2017	EEG: whole scalp; fNIRS: prefrontal	Simulated driving	EEG: beta power fNIRS: HbO	HbO and beta band-power in the frontal region detected drowsiness more rapidly than did eye-blinking.

## Studies of Multi-Modal Integration of EEG-fNIRS

Although visual EEG-BCI systems work well and demonstrate high reliability and performance, motor imagery EEG-BCI still suffers from low performance (far lower than 70% accuracy, which is commonly acceptable in EEG-BCI) and inter-subject variation (Ahn and Jun, 2015). Reportedly, some users intrinsically do not produce classifiable sensorimotor rhythms (Blankertz et al., 2010) or produce artifacts and ambient noise, neither of which can be addressed easily by mathematical algorithms. Some biological noise, such as eye-blinking or muscle movements, can be removed with elaborate preprocessing steps, but individual variation caused by different brain structures still may degrade BCI performance. Consequently, an optical BCI that uses fNIRS has been introduced (Coyle et al., 2004), and since then, a number of studies has been conducted on this technique (Naseer and Hong, 2015). In addition, numerous multi-modal approaches (Dornhege et al., 2004; Pfurtscheller et al., 2010) have been shown to improve BCI performance successfully.

For these reasons, the first study of EEG-fNIRS BCI was conducted to enhance performance of motor execution and imagery (Fazli et al., 2012). The authors collected EEG-fNIRS data simultaneously in the sensorimotor region, and 14 subjects were instructed to perform hand gripping and visual feedback-controlled motor imagery. They used Laplacian filtered band-power from EEG and HbO/HbR from fNIRS as features. An individual Linear discriminant analysis (LDA) classifier was computed first for EEG, HbO, and HbR, followed by a meta-classifier. They found that simultaneous EEG-fNIRS improved classification by 5% on average compared to a single modality. This was the first study of the potential use of EEG-fNIRS in BCI. A similar study was performed to decode motor imagery of the force and speed of hand clenching (Yin et al., 2015). They collected EEG-fNIRS data simultaneously in the sensorimotor region, and six subjects were asked to perform motor imagery using different forces and speeds of hand clenching for 10 s. Band-power, amplitude, phase, and frequency were combined to construct a time-phase-frequency feature from EEG, and the difference between HbO and HbR was extracted as a fNIRS feature. Importantly, they developed a feature optimization method using joint mutual information (Meyer et al., 2008) to remove redundant information that may reduce classification accuracy. Thereafter, they classified signals using the extreme learning machine (Huang et al., 2012). They achieved improved performance (up to a 5% increase) in decoding motor imagery of hand clenching by adopting a combination of EEG-fNIRS features.

It is known well that feedback training can improve BCI performance (Nyberg et al., 2006; Gentili et al., 2010). Specifically, patients with spinal cord injury showed preserved activation in the sensorimotor cortex after long-term training (Enzinger et al., 2008). However, little is known about the way in how BCI training influences brain activity and plastic changes over multiple training sessions. One study investigated these changes in the brain using multi-channel fNIRS with multiple visual feedback EEG-BCI training sessions (Kaiser et al., 2014). They designed an experimental paradigm that consisted of alternate fNIRS and EEG training sessions. All data were collected in the sensorimotor cortex and 15 subjects were asked to perform motor imagery (right hand and both feet) in all sessions. They found that training with the visual feedback BCI increased HbO in low BCI performers (<70%) accompanied by strong beta activity in EEG over sessions. This study demonstrated the way in how visual feedback EEG-BCI training affects brain activations associated with hemodynamic responses using fNIRS.

Inspired by the successful classification of mental arithmetic using fNIRS (Verner et al., 2013), one study attempted to decode mental arithmetic and motor execution from fNIRS and EEG, respectively (Khan et al., 2014). They designed an experimental paradigm that consisted of performing four different tasks for 60 s and calculated the classification accuracies between each task period and baseline. EEG and fNIRS sensors were placed on the sensorimotor and prefrontal regions, respectively. They achieved high classification accuracies (>80%) for all four tasks that can be applied in BCI systems.

The major limitation of fNIRS BCI is the inherent delay of the hemodynamic response, which makes it difficult to construct real-time BCI applications. In a previous study (Fazli et al., 2012), peak classification accuracy in fNIRS was delayed up to 7 s compared to that in EEG. Therefore, to overcome and adjust for this inherent delay, a recent study developed a new feature referred to as a slope indicator, which is the difference between the current time segment average and that computed from a previous time segment (Buccino et al., 2016). Band-power and HbO/HbR were used as inputs for LDA classifiers and 15 subjects were asked to perform four motor movements (left/right arm or hand). For all four tasks, EEG-fNIRS achieved higher classification accuracy compared to single modality and the slope indicator as a new feature in fNIRS reduced the delay in peak performance up to 2 s from onset. In addition to sensorimotor tasks, visual and auditory perception could be classified with high accuracy (>90%) using simultaneous EEG and fNIRS (Putze et al., 2014). This study suggested that passive BCI used to detect perceptual activity is also feasible for more natural BCI from the perspective of human–computer interaction.

A self-paced (asynchronous) BCI is able to discriminate between ongoing brain activity and that generated intentionally and reduce error rates as well. Some studies have used fNIRS features as a brain switch to design self-paced BCI. Koo et al. (2015) employed a novel experimental paradigm to detect the occurrence of motor imagery with fNIRS data. Threshold-based detection with a feature value of fNIRS data determined whether or not the action of a motor imagery task was attempted. Improving the performance of motor imagery is a primary issue in multi-modal integration of EEG-fNIRS, as performance using a single modality is relatively inferior to that in other BCI tasks. In addition to motor imagery, Tomita et al. (2014) merged two modalities in a steady-state visual evoked potential (SSVEP) BCI, which is the paradigm known best. They used an fNIRS signal in the occipital region as a brain switch to activate the SSVEP BCI. They extracted features from EEG and fNIRS, and classified SSVEP using a joint classifier. They achieved some improvement in SSVEP classification and reduced 13 subjects’ error rates. Although SSVEP BCI has worked well, designing self-paced BCI systems is essential to reduce the error rate, and to consider a subject’s intention in more naturalistic BCI.

Generally, EEG signals measured on the scalp consist of a mixture of neuronal activity that originates from different cortical areas and is contaminated inherently by background noise. To overcome this, various cortical current estimation methods have been developed (Grech et al., 2008). However, estimation of source current is an ill-posed inverse problem; thus, some prior assumptions are required to obtain a unique solution. Recently, hierarchical Bayesian estimation has been proposed to combine fMRI (Sato et al., 2004; Yoshioka et al., 2008) and fNIRS (Aihara et al., 2012) data as prior information. Consistent with these approaches, Morioka et al. (2014) employed fNIRS data as prior information for EEG cortical source estimation in BCI. Eight subjects were asked to perform a spatial attention task and normalized *t*-values (attention vs. control) from HbO features were used as the hierarchical prior information. They decoded directions (left or right) of spatial attention using a sparse logistic regression classifier based on cortical current sources and found an average 8% improvement in performance compared to EEG alone. Although this must be optimized well with respect to the number of dipoles or computational time for real-time BCI, this is the first study to use fNIRS data as prior information, which overcomes the inherent delay after task onset.

In addition to BCI studies, simultaneous EEG-fNIRS can be feasible in other fields. For example, monitoring long-term driving is quite suitable to determine drivers’ complex mental states and overcome delays in hemodynamic responses. Therefore, researchers have monitored performance during long-term simulated driving (Ahn et al., 2016; Nguyen et al., 2017) by using a custom-built fNIRS device compatible with a commercial EEG system to integrate the features from the two different modalities. They found that feature integration has great potential in monitoring driving performance while in a drowsy state, and investigated the interaction overall between two different features. Finally, they proposed neurophysiological correlates for monitoring good driving states using two non-invasive, portable techniques.

## Current Limitations and Future Directions

We reviewed studies that employed multi-modal integration of EEG-fNIRS for BCI. Most BCI studies have used each feature as an input for classifiers and achieved improvement in BCI performance. However, in all studies, this improvement (<10%) was modest compared to that obtained with a single modality. The ultimate purpose of multi-modal integration in BCI is to improve BCI performance, but this remains in its infancy. We may assume that the modest improvement is caused by the lack of computational approaches to feature integration and the mismatch in the recording locations between the two modalities. Therefore, in the sections that follow, we discuss current limitations and future directions for successful multi-modal integration of EEG-fNIRS.

### Integration of Two Different Features

To date, HbO and HbR are unique features of fNIRS recordings, and have been used widely. Because HbO and HbR are both temporal features, it is important in signal processing to determine the temporal period and apply filtering methods based on the temporal domain. In contrast, both spectral and temporal features can be used in EEG, and thus, subjects often are presented with rapid and short stimuli. However, such stimuli are not applicable in fNIRS recordings because of the inherent response delays and low temporal resolution. An fNIRS system measures hemodynamic responses, which take several seconds to develop. Delays in hemodynamic responses have been estimated with modeling simulations and computational methods (Liao et al., 2002; Buxton et al., 2004). An invasive approach also has demonstrated the delays in hemodynamic responses (Duckett et al., 2011). Therefore, temporal synchronization for different temporal resolutions and measurement delays between fNIRS and EEG must be addressed carefully before simultaneous recordings. In BCI systems in particular, temporal synchronization could be a critical problem because the information transfer rate is the most important factor used to assess BCI systems. To handle these problems, computational methods, such as using prior information (Morioka et al., 2014) or normalized features (Ahn et al., 2016), are necessary to obtain BCI performance better than that with a single modality. Morioka et al. (2014) used fNIRS features as prior information to estimate cortical current in EEG, while Ahn et al. (2016) combined EEG and fNIRS features by normalizing all features that ranged from 0 to 1, and applying a summation of the features. Although further optimization steps still are required, these two novel approaches may be future solutions that overcome the current limitations in integrating the features of EEG-fNIRS.

### Sensor Configuration of Two Different Devices

**FIGURE 1:**
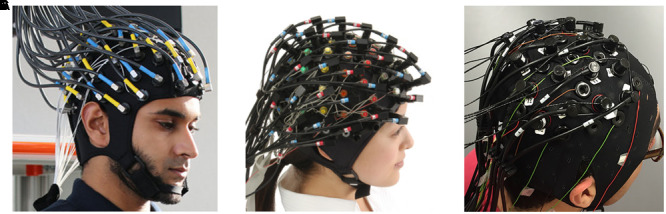
Commercial EEG-fNIRS devices: **(A)** fNIRS-EEG package (Courtesy of Artinis Medical Systems, Netherlands, http://www.artinis.com, up to 112-ch fNIRS and 128-ch EEG); **(B)** LABNIRS (Courtesy of Shimadzu Corporation, Japan, http://www.shimadzu.com, up to 142-ch fNIRS and 64-ch EEG); **(C)** NIRScout (Courtesy of NIRx Medical Technologies, http://nirx.net, Up to 182-ch fNIRS and 32-ch EEG) created by Buccino et al. (2016).

Although there are several commercial devices on the market that allow simultaneous measurement of EEG-fNIRS (Figure 1), it is difficult to record neuronal activity from the same location. Because hemodynamic responses are recorded from the middle of emitters and detectors in fNIRS, EEG electrodes should be placed in the middle of these emitter and detectors to achieve the same channel configuration. EEG electrodes can be very small, but emitters and detectors in fNIRS devices must be relatively sizable to emit and detect infrared lights properly. In addition, the quantification of emitted and detected infrared light is affected substantially by dense hair (Gervain et al., 2011). Dense hair may generate a low signal-to-noise ratio in fNIRS measurements such that it is difficult to measure sensitive hemodynamic changes in the brain. These technical problems also hinder same-channel configuration and the ability to cover the entire brain region. One possible way to equip an EEG-fNIRS device in the laboratory is to combine them manually without a well-tuned hardware combination. One study (Koo et al., 2015) designed a blocking frame made of black acrylic plastic with a compression rubber pad and obtained fNIRS and EEG data simultaneously. In addition, researchers have custom-built compact and wireless EEG-fNIRS devices (Safaie et al., 2013; Sawan et al., 2013; von Lühmann et al., 2015, 2017). Such hardware developments may enable us to resolve current technical limitations to some extent.

## Conclusion

In this work, we reviewed studies of the multi-modal integration of EEG-fNIRS. In our literature review, we found that BCI using EEG-fNIRS has considerable potential to improve performance by measuring two different brain activities. However, it suffers from two major problems: the lack of computational methods to integrate the features and optimized sensor configuration. We suggested possible ways to overcome the current limitations based on previous work. Computational integration methods should be developed that consider the characteristics of features from multi-modal EEG/fNIRS signals. Further, sophisticated hardware developments are needed that address the size of sensors and light leakage in fNIRS. We believe that our review and suggestions for multi-modal integration can be a stepping stone in making a significantly advanced brain measurement tool with better portability than EEG or fNIRS alone.

## Author Contributions

SA did literature surveys. SA and SJ discussed all findings and wrote this manuscript. SJ coordinated all necessary procedures.

## Conflict of Interest Statement

The authors declare that the research was conducted in the absence of any commercial or financial relationships that could be construed as a potential conflict of interest.

## Footnotes

1 www.intendix.com
