# Corrigendum: Multi-Modal Integration of EEG-fNIRS for Brain-Computer Interfaces – Current Limitations and Future Directions

**DOI:** 10.3389/fnhum.2021.645869

**Published:** 2021-02-01

**Authors:** Sangtae Ahn, Sung C. Jun

**Affiliations:** ^1^School of Electronic and Electrical Engineering, Kyungpook National University, Daegu, South Korea; ^2^School of Electronics Engineering, Kyungpook National University, Daegu, South Korea; ^3^School of Electrical Engineering and Computer Science, Gwangju Institute of Science and Technology, Gwangju, South Korea

**Keywords:** multi-modal integration, electroencephalography (EEG), functional near-infrared spectroscopy (fNIRS), brain-computer interface (BCI)

In the original article, there was an error. Sentences include misinformation.

A correction has been made to ^**^CURRENT LIMITATIONS AND FUTURE DIRECTIONS^**^, ^**^Sensor Configuration of Two Different Devices^**^, ^**^First Paragraph^**^

**ORIGINAL SENTENCES**

“However, because light leakage from an fNIRS system can contaminate EEG signals, it is necessary to block this leakage when combining the two devices. One study (Koo et al., 2015) designed a blocking frame made of black acrylic plastic with a compression rubber pad. They blocked the light that leaked from emitters successfully and obtained unaffected EEG signals.”

**CORRECTED AS FOLLOWS**

“One study (Koo et al., 2015) designed a blocking frame made of black acrylic plastic with a compression rubber pad and obtained fNIRS and EEG data simultaneously”

The authors apologize for this error and state that this does not change the scientific conclusions of the article in any way. The original article has been updated.
